# Alcoholic liver disease-from steatosis to cirrhosis - a biochemistry approach

**DOI:** 10.25122/jml-2021-0081

**Published:** 2021

**Authors:** Ruxandra Cioarca-Nedelcu, Valeriu Atanasiu, Irina Stoian

**Affiliations:** 1. Biochemistry Department, University of Medicine and Pharmacy Carol Davila, Bucharest, Romania

**Keywords:** ethanol, oxidative stress, steatosis, steatohepatitis, cirrhosis, ADH – alcohol dehydrogenase, ALDH2 – aldehyde dehydrogenase 2, CYP2E1 – cytochrome P450 2E1, ER – endoplasmic reticulum, NAD^+^ – nicotinamide adenine dinucleotide, NADH – reduced nicotinamide adenine dinucleotide, ROS – reactive oxygen species, DNA – deoxyribonucleic acid, NADP^+^ – nicotinamide adenine dinucleotide phosphate, NADPH – reduced nicotinamide adenine dinucleotide phosphate, O_2_^-^∙ – superoxide anion, H_2_O_2_ – hydrogen peroxide, -OH – hydroxyl radical, HER – hydroxyethyl radical, GSH – glutathione, A(2)C – 2-(2-methoxy ethoxy) ethyl 8-(cis-2-n-octylcyclopropyl), MAA – malondialdehyde-acetaldehyde compounds, TNFalpha – tumor necrosis factor-alpha, AMPK – adenosine monophosphate-activated protein kinase, PPARalpha – peroxisome proliferator-activated receptor-alpha, SREBP-1 – sterol regulatory element-binding protein 1, HSC – hepatic stellate cells, KC – Kupffer cells, LPS – lipopolysaccharides, TLR4 – toll-like receptor 4, CD14 – cluster of differentiation, JNK – Janus Kinase

## Abstract

Nowadays, chronic alcoholism and its health implications represent a global concern. Over three million deaths are linked to chronic alcohol intake every year. This article aims to spread awareness about the negative impact ethanol can have on almost every organ in the body, especially the liver. Understanding ethanol metabolism and the cellular pathways through which alcohol increases liver oxidative stress may prevent a broad spectrum of hepatic lesions such as steatosis, steatohepatitis, and, ultimately, cirrhosis. After a short review of ethanol metabolism and liver oxidative stress, each hepatic lesion will be individually discussed regarding the mechanism of apparition, treatment, and future targeted therapies.

## Introduction

Chronic alcoholism represents a worldwide concern with a vast social and economic impact on the healthcare systems. Globally, approximately 3 million deaths are registered as alcohol-related every year [1]. In addition, excessive ethanol intake over several decades can have a negative impact on almost every organ in the body [2]. However, the liver, the main site of alcohol metabolism, is the organ that suffers the most extensive tissue injury [3].

After a short review of ethanol metabolism in the liver, this article will summarize the cellular pathways through which alcohol induces liver oxidative stress and determines a broad spectrum of hepatic lesions such as steatosis, steatohepatitis, and ultimately cirrhosis [4].

## Material and Methods

The PubMed database was searched by selecting articles from the past 20 years and using keywords such as ethanol, oxidative stress, steatosis, steatohepatitis, and cirrhosis. Also, words such as AND and OR were used as combining terms, enabling us to limit the information. The review and original articles resulting from our search were selected in conformity with the main purpose of this study.

## Results

### Alcohol metabolism in the liver- a biochemistry approach

Ethanol is mainly metabolized in hepatocytes, the parenchymal cells of the liver. Hepatocytes contain important levels of enzymes that are involved in ethanol oxidation: alcohol dehydrogenase (ADH), situated in the cytosol, aldehyde dehydrogenase 2 (ALDH2) [5], situated in the mitochondria, and cytochrome P450 2E1(CYP2E1), situated in the endoplasmic reticulum (ER) [6]. Moreover, hepatocytes also contain high amounts of catalase, an enzyme found in peroxisomes. Catalase breaks down hydrogen peroxide transforming it into water and oxygen. If present, ethanol activates a secondary function of catalase that oxidizes ethanol to acetaldehyde using hydrogen peroxide (H_2_O_2_) [3].

ADH, one of the major hepatic enzymes that catalyses ethanol oxidation, needs cofactor nicotinamide adenine dinucleotide (NAD^+^) to generate reduced NAD^+^(NADH) and acetaldehyde – an extremely toxic compound [7]. Because of its electrophilic nature, acetaldehyde can covalently bind to proteins, lipids, and nucleic acids and form acetaldehyde adducts that alter intracellular homeostasis [8].

Cellular proteins become highly susceptible to proteolysis, changes in their electric charge, and enzyme inactivation. In order to reduce toxicity, acetaldehyde is rapidly further oxidized to acetate by enzyme ALDH2 inside mitochondria. This reaction generates acetate and even more NADH [9].

As a result, the cellular NAD^+^/NADH ratio decreases, having a strong impact on the inhibition of fatty acid beta-oxidation, causing triacylglycerols to accumulate inside hepatocytes [10]. Besides its important role in the development of liver steatosis, massive generation of NADH has other significant effects on the citric acid cycle, gluconeogenesis, and glycolysis. NADH is reoxidized to NAD^+^ by the electron transfer chain in the mitochondria [7]. While electrons are transferred towards oxygen in the inner mitochondrial membrane, a high amount of reactive oxygen species (ROS) such as the hydroxyl radical (OH), superoxide anion (O_2_^-^∙), and hydrogen peroxide (H_2_O_2_) is formed [11].

Regarding nucleic acids, both nuclear and mitochondrial deoxyribonucleic acid (DNA) are vulnerable in front of ROS [12]. Any impairment in the genetic code will affect the rightful synthesis of cellular proteins. Several studies have shown that 8-oxo-guanine is the primary marker of alcohol-induced DNA damage. Its level has been measured in the liver of rats after chronic alcohol administration [13].

CYP2E1 is the other important liver enzyme located mainly in the endoplasmic reticulum. In the presence of molecular oxygen, CYP2E1 oxidates ethanol to acetaldehyde and transforms reduced NAD phosphate (NADPH) to NADP+, producing water [14]. CYP2E1 can increase its levels as an inducible enzyme, especially during chronic alcohol consumption [15]. Whenever CYP21E1 exceeds its normal level, molecular oxygen becomes an important substrate for the enzyme, displaying a high amount of reactive oxygen species such as superoxide anion (O_2_^-^∙), hydrogen peroxide (H_2_O_2_), hydroxyl radical (-OH), and the hydroxyethyl radical (HER) [16]. Out of the microsomal cytochrome P-450 enzymes, CYP2E1 is the most susceptible to producing ROS because of its weak connection with the CYP redox cycle and with NADPH-cytochrome P450 reductase [17].

Repeated formation of these reactive oxygen species, especially in heavy drinkers, surpasses the liver’s capacity of fighting oxidative stress by consuming all resources of the local enzymatic and non-enzymatic antioxidant molecules [18].

The main non-enzymatic molecules are vitamin A, vitamin C, vitamin E, bilirubin, and glutathione (GSH) [19]. GSH, the most important non-enzymatic antioxidant, has been depleted in mitochondria after chronic ethanol intake. GSH is highly necessary for mitochondria to remove ROS generated in the respiratory chain. Bailey *et al.* proved that mitochondrial depletion of GSH in chronic alcoholism is due to an impairment of GSH transport from cytosol to the mitochondrial matrix through the 2-oxoglutarate carrier. Molecules such as 2-(2-methoxy ethoxy) ethyl 8-(cis-2-n-octylcyclopropyl) (A(2)C) were able to correctly restore GSH transport [20].

Dominant hepatic antioxidant enzymes are glutathione reductase, glutathione peroxidase, catalase (involved in H_2_O_2_ removal), and superoxide dismutase (involved in superoxide anion disposal) [19].

Liver oxidative stress becomes more accentuated when the already generated reactive oxygen species are involved in auxiliary reactions with unsaturated lipids, generating lipid peroxides [21]. Lipid peroxides will further react with acetaldehyde and proteins, forming larger compounds such as malondialdehyde-acetaldehyde compounds (MAA). These MAA compounds can induce a strong inflammatory response in the liver, also known as alcoholic hepatitis [8].

Nowadays, multiple studies targeting alcohol-induced liver disease have shown that administration of agents that eradicate superoxide anions, such as a superoxide dismutase [22] and agents that restore reduced glutathione, such as N-acetyl cysteine [23], can successfully counteract oxidative stress. Moreover, through their anti-inflammatory activity, corticosteroids can decrease the local release of cytokines and the local collagen production, especially in alcoholic hepatitis [24]. Last but not least, silymarin, which is a strong antioxidant, is a strong cell membrane stabilizer and an antifibrotic agent [25].

### Alcoholic induced steatosis

Hepatic steatosis, also known as fatty liver disease, represents the accumulation of intrahepatic triacylglycerols in a minimum of 5% of the total liver weight. Steatosis is the primary and the most encountered response when the liver is exposed to chronically high levels of ethanol. It is considered a benign and reversible condition that ceases whenever the subject stops drinking. From a histopathological point of view, steatosis starts as the accumulation of lipid droplets inside the perivenular hepatocytes, expands towards the mid-lobular hepatocytes, and ultimately progresses to the periportal hepatocytes [26]. The main pathways involved in the development of alcohol-induced hepatic steatosis will be discussed further on.

Ethanol, through ROS, acetaldehyde, and tumor necrosis factor-alpha (TNFalpha) generation, impairs hepatic lipid metabolism. As a result, lipolysis and fatty acids beta-oxidation decrease in favour of lipogenesis and cholesterol synthesis. Ethanol, together with ROS, acetaldehyde, and TNFalpha, are directly responsible for the inhibition of adenosine monophosphate-activated protein kinase (AMPK). This molecule plays a central role in the accumulation of triacylglycerols inside hepatocytes.

By inhibiting AMP-activated PK, ethanol downregulates peroxisome proliferator-activated receptor alpha (PPARalpha), decreasing beta-oxidation and increasing local inflammation through the NF-kB p65 signaling system [27]. Several studies have shown that molecule imipramine can cancel AMPK inhibition by being a strong inactivator of the ASMase enzyme [28]. Moreover, fibrates, as low activators of the PPARalpha transcription factor, can prevent and treat hepatic steatosis [29].

Another ethanol-impaired signaling pathway involved in hepatic steatosis is represented by the activation of sterol regulatory element-binding protein 1 (SREBP-1). Normally, SREBPc-1 is inactive in hepatocytes of non-alcoholic subjects. However, hepatic ethanol exposure sets off the translocation of SREBP-1 from the endoplasmic reticulum to the Golgi apparatus, where it maturates through proteolysis. Maturated SREBP-1 then launches into the nucleus, where it stimulates transcription of lipogenic genes. As a result, SREBP-1 strongly increases intrahepatocytar fatty acid synthesis through fatty acid synthase and stearoyl-Coenzyme A desaturase activation [30]. Blockers of SREBP-1 activation are highly used molecules nowadays, such as ursodeoxycholic acid [31] and resveratrol [32].

In addition to the outcomes described above, AMPK inhibition also determines a decrease in fatty acids beta-oxidation by acting on acetyl Coenzyme A carboxylase and carnitine palmitoyltransferase [33]. In conclusion, ethanol-induced AMPK inhibition represents a central point in ethanol-induced hepatic steatosis through SREBP-1 activation and PPAR-alpha downregulation.

Another important role in ethanol-induced steatosis is represented by insulin resistance. Numerous studies have shown an accelerated evolution of ethanol-induced liver disease (from steatosis to steatohepatitis) in alcoholic obese patients compared to normal-weight alcoholic patients. In addition, studies on ethanol-fed mice proved that alcohol determines macrophage infiltration, cytokine releasing of TNF alpha and Interleukine 6, and insulin resistance in adipose tissue. Also, an important reduction of adiponectin in adipose tissue has been discovered, apparently due to endoplasmic reticulum injury. Low local adiponectin levels can directly reduce fatty acid oxidation and promote fatty acid production through AMPK inhibition [34].

### Alcoholic steatohepatitis

Alcoholic steatohepatitis is a chronic liver disease characterized by fatty liver inflammation, which usually occurs in chronic alcohol consumers. Pathologic indicators of alcoholic hepatitis are ballooning of hepatocytes, intrahepatocytar Mallory bodies, local infiltration of neutrophils, and fibrosis [24].

The key point in ethanol-induced hepatitis is represented by local macrophages, also known as Kupffer cells. Kupffer cells are located in liver sinusoids and act as a primary defense line whenever the liver is exposed to pathogens and toxic compounds via the portal venous tract [35].

Physiologically, Kupffer cells do not respond to all antigens with an immune response. However, in subjects with chronic alcoholism, Kupffer cells can switch from an anti-inflammatory to a strong pro-inflammatory state. Activated Kupffer cells generate a significant amount of free radical species through the phagocytic type of NADPH oxidase and CYP2E1enzymatic systems [36]. Generated reactive oxygen and nitrogen species stimulate the release of several cytokines such as TNF alpha, chemokines, and interleukins that ultimately enhance the local attraction of polymorphonuclear cells from the bloodstream into the liver parenchyma [37].

The main trigger of Kupffer cells’ immune response in subjects with chronic ethanol consumption is an endotoxin known as lipopolysaccharide (LPS). This LPS represents a part of a Gram-negative bacteria’s wall usually found in normal colonic microbiota [38]. Several studies have shown that chronic ethanol intake is responsible for bacterial translocation from the gut to the liver through the spleno-portal axis. This process, also known as endotoxemia, occurs because of alcohol’s ability to increase intestinal permeability, promoting dysbiosis and local bacterial overgrowth [39].

Alcohol is directly responsible for the destruction of the tight interepithelial junctions that interconnect intestinal cells. Therefore, chronic alcoholism can increase intestinal permeability and allow pathogenic bacteria and endotoxins into the portal circulation. This process is known in the literature as a “leaky gut” [40]. In chronic alcoholism, intestinal microbiota also suffers a critical imbalance in the ratio between beneficial bacteria such as Lactobacillus and Bifidobacterium and harmful bacteria such as proteobacteria and bacilli.

In addition, alcohol is a strong suppressor of T cells activity, impairing the intestinal mucosal response and pathogenic bacteria cleareance [41].

Due to all of the mechanisms explained above, LPS can reach the liver, get detected by KCs’ receptors – CD14 and toll-like receptor 4 (TLR4), chemoattract cytotoxic neutrophils, and activate NADPH oxidase in Kupffer cells. As a result, ROS generation and tumor necrosis factor-alpha (TNFalpha) synthesis is increased, inducing local liver inflammation and injury [42]. In conclusion, there is a strong connection between high endotoxemia and the progression of alcoholic liver disease from liver steatosis to alcoholic hepatitis.

Nowadays, two of the most studied molecules to prevent and reverse alcohol-induced steatohepatitis are Diphenyleneiodonium (inhibitor of NADPH oxidase) [43] and di-linoleoyl phosphatidylcholine (antioxidant) [44].

### Cirrhosis

Cirrhosis represents the final stage of liver fibrosis, which in the majority of cases is caused by underlying conditions such as viral B or C hepatitis and chronic alcoholism. It is histologically represented by excessive accumulation of extracellular matrix, mainly fibrillar collagen, into the liver parenchyma, leading to various structural and functional abnormalities [45].

There are numerous types of cells involved in the development of alcoholic cirrhosis. The most important ones are hepatic stellate cells (HSC) and Kupffer cells.

Hepatic stellate cells are located in the space of Disse, and their main function is to store vitamin A as retinyl esters. Subsequently to chronic alcoholism, there is a significant depletion of vitamin A in HSC, leading to a switch in the HSC phenotype. HSC will turn into a myofibroblast pro-fibrogenic-like type of cells, increasing their quantity of rough endoplasmic reticulum and their expression of alpha-smooth muscle actin [46].

Further on, activated HSC will generate pro-inflammatory cytokines and extracellular matrix proteins, mainly type I collagen, participating in the progression of alcoholic liver disease from fibrosis to cirrhosis [47].

Kupffer cells, by releasing profibrogenic cytokines (tumor growth factor-beta and platelet-derived growth factor), also play an essential role in the activation of the non-phagocytic type of NADPH oxidase in HSC. In response, HSC will generate even more local ROS, increasing local inflammation, tissue injury, and fibrotic healing [48].

Several studies have shown that ROS, generated as part of Kupffer cells and HSC activity, can increase liver fibrosis by activating several fibrosis-associated hepatic genes through Janus Kinase (JNK) and NFkB pathways. The most important genes are inhibitor of metalloproteinase-1 gene, monocyte chemoattractant protein-1 gene, and procollagen Type 1 gene [49].

### Promising treatments for liver fibrosis that target oxidative stress in the endoplasmic reticulum and mitochondria

Nowadays, oxidative stress is seen as an important tool in liver fibrosis treatment. Even though antioxidants (such as vitamins) have been conventionally used for decades as unspecific alleviators of ROS buildup, their therapeutic effect for several pathologies, including liver fibrosis, remains questionable. Drugs that aim for ROS generators, such as mitochondrial dysfunction and endoplasmic reticulum stress, are novel therapies that will be discussed further on [50].

### Endoplasmic reticulum inhibitors

The main role of the endoplasmic reticulum is represented by protein folding. The unfolded protein response is activated whenever too many misfolded or unfolded proteins are generated through the endoplasmic reticulum. This response is responsible for destroying and/or repairing deformed or unfolded proteins. Whenever this type of response is required to deal with a large quantity of misfolded proteins (e.g. chronic alcohol consumption), excessive H_2_O_2_ forms, leading to increased oxidative stress and apoptosis [51]. Ero1α and PDI (protein disulfide isomerase) are the main enzymes that create disulfide bonds in proteins during the endoplasmic reticulum processing. The impaired activity of these enzymes, together with decreased levels of GSH and dysfunction of electron transport chain proteins, play a crucial role in generating liver fibrosis, especially in alcoholic liver disease [52]. Inhibiting Ero1α with a specific molecule: EN460, showed defensive effects in artificially induced endoplasmic reticulum stress [53]. Other inhibitor molecules such as GSK2600414 and GSK2656157 that target PERK (Protein kinase R (PKR)-like endoplasmic reticulum kinase), a local oxidative stress sensor, showed a significant reduction in the unfolded protein response and the generation of the oxidative stress [50].

### Mitochondrial dysfunction inhibitors

Alcoholic liver disease has been associated with the loss of mitochondrial function, primarily by impairing the electron transport chain and permeability of the inner mitochondrial membrane, resulting in excessive H_2_O_2_ formation. Several studies on mice and rats have shown that Coenzyme Q10, one of the major electron transport chain components, can decrease liver fibrosis and oxidative stress when administered orally. Another inhibitor of mitochondrial dysfunction that modulates mytosol calcium homeostasis and regulates inner mitochondrial membrane permeability is NIM811. In a rat model study, NIM811 decreased the expression fibrosis of markers in HSC cells and urged liver recovery [54].

## Conclusion

Up-to-the present, several researchers have demonstrated that alcohol impacts the genesis of all entities included in the alcohol liver disease definition. The importance of understanding the mechanisms through which ethanol increases liver oxidative stress and reduces the local antioxidant barrier have been the subject of several studies, thus providing hope for future therapeutic strategies.

## Acknowledgments

### Conflict of interest

The authors declare that there is no conflict of interest.

### Personal thanks

We would like to thank Irina Stoian, Professor at the Biochemistry Department of Carol Davila University of Medicine and Pharmacy, Bucharest, for assisting us in the execution of the review and the preparation of the manuscript.

### Authorship

R.C. was responsible for data acquisition and for the writing of the manuscript. I.S. together with V. A. were responsible for the concept and for the proofreading of the manuscript.

## References

[1] Mann RE, Smart RG, Govoni R (2003). The epidemiology of alcoholic liver disease. Alcohol Res Heal.

[2] Lieber CS (2000). Alcohol: Its metabolism and interaction with nutrients. Annu Rev Nutr.

[3] Gramenzi A, Caputo F, Biselli M, Kuria F, Loggi E, Andreone P, Bernardi M (2006). Review article: Alcoholic liver disease - Pathophysiological aspects and risk factors. Aliment Pharmacol Ther.

[4] Louvet A, Mathurin P (2015). Alcoholic liver disease: Mechanisms of injury and targeted treatment. Nat Rev Gastroenterol Hepatol.

[5] Crabb DW, Matsumoto M, Chang D, You M (2004). Overview of the role of alcohol dehydrogenase and aldehyde dehydrogenase and their variants in the genesis of alcohol-related pathology. Proc Nutr Soc.

[6] Lu Y, Cederbaum AI (2008). CYP2E1 and oxidative liver injury by alcohol. Free Radic Biol Med.

[7] Meijers R, Morris RJ, Adolph HW, Merli A, Lamzin VS, Cedergren-Zeppezauer ES (2001). On the Enzymatic Activation of NADH. J Biol Chem.

[8] Setshedi M, Wands JR, De La Monte SM (2010). Acetaldehyde adducts in alcoholic liver disease. Oxid Med Cell Longev.

[9] Van Vleet TR, Philip BK (2014). Acetaldehyde. Encycl Toxicol Third Ed.

[10] Lieber CS (2004). Alcoholic fatty liver: Its pathogenesis and mechanism of progression to inflammation and fibrosis. Alcohol.

[11] Wu D, Cederbaum AI (2009). Oxidative stress and alcoholic liver disease. Semin Liver Dis.

[12] Hoek JB, Pastorino JG (2002). Ethanol, oxidative stress, and cytokine-induced liver cell injury. Alcohol.

[13] Hirano T (2011). Alcohol consumption and oxidative DNA damage. Int J Environ Res Public Health.

[14] Gonzalez FJ (2007). The 2006 Bernard B. Brodie award lecture CYP2E1. Drug Metab Dispos.

[15] Linhart K, Bartsch H, Seitz HK (2014). The role of reactive oxygen species (ROS) and cytochrome P-450 2E1 in the generation of carcinogenic etheno-DNA adducts. Redox Biol.

[16] Bansal S, Liu CP, Sepuri NBV, Anandatheerthavarada HK, Selvaraj V, Hoek J, Milne GL, Guengerich FP, Avadhani NG (2010). Mitochondria-targeted cytochrome P450 2E1 induces oxidative damage and augments alcohol-mediated oxidative stress. J Biol Chem.

[17] Cederbaum AI (2006). CYP2E1 - Biochemical and toxicological aspects and role in alcohol-induced liver injury. Mt Sinai J Med.

[18] Wu D, Cederbaum AI (2003). Alcohol, oxidative stress, and free radical damage. Alcohol Res Heal.

[19] Ha HL, Shin HJ, Feitelson MA, Yu DY (2010). Oxidative stress and antioxidants in hepatic pathogenesis. World J Gastroenterol.

[20] Ribas V, García-Ruiz C, Fernández-Checa JC (2014). Glutathione and mitochondria. Front Pharmacol.

[21] Sozio M, Crabb DW (2008). Alcohol and lipid metabolism. Am J Physiol - Endocrinol Metab.

[22] Koch OR, Pani G, Borrello S, Colavitti R, Cravero A, Farrè S, Galeotti T (2004). Oxidative stress and antioxidant defenses in ethanol-induced cell injury. Mol Aspects Med.

[23] Mokhtari V, Afsharian P, Shahhoseini M, Kalantar SM, Moini A (2017). A review on various uses of N-acetyl cysteine. Cell J.

[24] Stickel F, Seitz HK (2010). Alcoholic steatohepatitis. Best Pract Res Clin Gastroenterol.

[25] Federico A, Dallio M, Loguercio C (2017). Silymarin/Silybin and chronic liver disease: A marriage of many years. Molecules.

[26] El-Zayadi AR (2008). Hepatic steatosis: A benign disease or a silent killer. World J Gastroenterol.

[27] Sid B, Verrax J, Calderon PB (2013). Role of AMPK activation in oxidative cell damage: Implications for alcohol-induced liver disease. Biochem Pharmacol.

[28] Liangpunsakul S, Sozio MS, Shin E, Zhao Z, Xu Y, Ross RA, Zeng Y, Crabb DW (2010). Inhibitory effect of ethanol on AMPK phosphorylation is mediated in part through elevated ceramide levels. Am J Physiol - Gastrointest Liver Physiol.

[29] Staels B, Maes M, Zambon A (2008). Fibrates and future PPARα agonists in the treatment of cardiovascular disease. Nat Clin Pract Cardiovasc Med.

[30] You M, Crabb DW (2004). Molecular mechanisms of alcoholic fatty liver: Role of sterol regulatory element-binding proteins. Alcohol.

[31] Hu J, Zhu XH, Ye L, Hong W, Yao KN, Chen ZY (2019). Ursodeoxycholic acid ameliorates hepatic lipid metabolism in LO2 cells by regulating the AKT/mTOR/SREBP-1 signaling pathway. World J Gastroenterol.

[32] Wang GL, Fu YC, Xu WC, Feng YQ, Fang SR, Zhou XH (2009). Resveratrol inhibits the expression of SREBP1 in cell model of steatosis via Sirt1-FOXO1 signaling pathway. Biochem Biophys Res Commun.

[33] Lally JSV, Ghoshal S, DePeralta DK, Moaven O, Wei L, Masia R, Erstad DJ, Fujiwara N, Leong V, Houde VP, Anagnostopoulos AE, Wang A, Broadfield LA, Ford RJ, Foster RA, Bates J, Sun H, Wang T, Liu H, Ray AS, Saha AK, Greenwood J, Bhat S, Harriman G, Miao W, Rocnik JL, Westlin WF, Muti P, Tsakiridis T, Harwood HJ, Kapeller R, Hoshida Y, Tanabe KK, Steinberg GR, Fuchs BC (2019). Inhibition of Acetyl-CoA Carboxylase by Phosphorylation or the Inhibitor ND-654 Suppresses Lipogenesis and Hepatocellular Carcinoma. Cell Metab.

[34] Ramirez T, Tong M, Chen WC, Nguyen QG, Wands JR, De la Monte SM (2013). Chronic alcohol-induced hepatic insulin resistance and endoplasmic reticulum stress ameliorated by peroxisome-proliferator activated receptor-δ agonist treatment. J Gastroenterol Hepatol.

[35] Kolios G, Valatas V, Kouroumalis E (2006). Role of Kupffer cells in the pathogenesis of liver disease. World J Gastroenterol.

[36] Wan J, Benkdane M, Teixeira-Clerc F, Bonnafous S, Louvet A, Lafdil F, Pecker F, Tran A, Gual P, Mallat A, Lotersztajn S, Pavoine C (2014). M2 Kupffer cells promote M1 Kupffer cell apoptosis: A protective mechanism against alcoholic and non-alcoholic fatty liver disease. Hepatology.

[37] Marra F, Tacke F (2014). Roles for chemokines in liver disease. Gastroenterology.

[38] Szabo G, Bala S (2010). Alcoholic liver disease and the gut-liver axis. World J Gastroenterol.

[39] Szabo G (2015). Gut-liver axis in alcoholic liver disease. Gastroenterology.

[40] Leclercq S, Cani PD, Neyrinck AM, Stärkel P, Jamar F, Mikolajczak M, Delzenne NM, De Timary P (2012). Role of intestinal permeability and inflammation in the biological and behavioral control of alcohol-dependent subjects. Brain Behav Immun.

[41] Forsyth CB, Farhadi A, Jakate SM, Tang Y, Shaikh M, Keshavarzian A (2009). Lactobacillus GG treatment ameliorates alcohol-induced intestinal oxidative stress, gut leakiness, and liver injury in a rat model of alcoholic steatohepatitis. Alcohol.

[42] Roh YS, Seki E (2013). Toll-like receptors in alcoholic liver disease, non-alcoholic steatohepatitis and carcinogenesis. J Gastroenterol Hepatol.

[43] Li Y, Trush MA (1998). Diphenyleneiodonium, an NAD(P)H oxidase inhibitor, also potently inhibits mitochondrial reactive oxygen species production. Biochem Biophys Res Commun.

[44] Lieber CS, Robins SJ, Jianjun L, DeCarli LM, Mak KM, Fasulo JM, Leo MA (1994). Phosphatidylcholine protects against fibrosis and cirrhosis in the baboon. Gastroenterology.

[45] Czaja AJ (2014). Hepatic inflammation and progressive liver fibrosis in chronic liver disease. World J Gastroenterol.

[46] Friedman SL (2008). Mechanisms of Hepatic Fibrogenesis. Gastroenterology.

[47] Tsuchida T, Friedman SL (2017). Mechanisms of hepatic stellate cell activation. Nat Rev Gastroenterol Hepatol.

[48] Paik YH, Brenner DA (2011). NADPH oxidase mediated oxidative stress in hepatic fibrogenesis. Korean J Hepatol.

[49] Richter K, Kietzmann T (2016). Reactive oxygen species and fibrosis: further evidence of a significant liaison. Cell Tissue Res.

[50] Rodríguez LR, Calap-Quintana P, Lapeña-Luzón T, Pallardó F V., Schneuwly S, Navarro JA, Gonzalez-Cabo P (2020). Oxidative stress modulates rearrangement of endoplasmic reticulum-mitochondria contacts and calcium dysregulation in a Friedreich’s ataxia model. Redox Biol.

[51] Bataller R, Brenner DA (2001). Hepatic stellate cells as a target for the treatment of liver fibrosis. Semin Liver Dis.

[52] Xia SW, Wang ZM, Sun SM, Su Y, Li ZH, Shao JJ, Tan SZ, Chen AP, Wang SJ, Zhang ZL, Zhang F, Zheng SZ (2020). Endoplasmic reticulum stress and protein degradation in chronic liver disease. Pharmacological Research.

[53] Hayes KE, Batsomboon P, Chen WC, Johnson BD, Becker A, Eschrich S, Yang Y, Robart AR, Dudley GB, Geldenhuys WJ, Hazlehurst LA (2019). Inhibition of the FAD containing ER oxidoreductin 1 (Ero1) protein by EN-460 as a strategy for treatment of multiple myeloma. Bioorganic Med Chem.

[54] Mantena SK, King AL, Andringa KK, Eccleston HB, Bailey SM (2008). Mitochondrial dysfunction and oxidative stress in the pathogenesis of alcohol- and obesity-induced fatty liver diseases. Free Radical Biology and Medicine.

